# Novel loss-of-function variants expand *ABCC9*-related intellectual disability and myopathy syndrome

**DOI:** 10.1093/brain/awae010

**Published:** 2024-01-13

**Authors:** Stephanie Efthymiou, Marcello Scala, Vini Nagaraj, Katarzyna Ochenkowska, Fenne L Komdeur, Robin A Liang, Mohamed S Abdel-Hamid, Tipu Sultan, Tuva Barøy, Marijke Van Ghelue, Barbara Vona, Reza Maroofian, Faisal Zafar, Fowzan S Alkuraya, Maha S Zaki, Mariasavina Severino, Kingsley C Duru, Robert C Tryon, Lin Vigdis Brauteset, Morad Ansari, Mark Hamilton, Mieke M van Haelst, Gijs van Haaften, Federico Zara, Henry Houlden, Éric Samarut, Colin G Nichols, Marie F Smeland, Conor McClenaghan

**Affiliations:** Department of Neuromuscular Disorders, UCL Queen Square Institute of Neurology, University College London, London WC1N 3BG, UK; Department of Neuromuscular Disorders, UCL Queen Square Institute of Neurology, University College London, London WC1N 3BG, UK; Department of Neurosciences, Rehabilitation, Ophthalmology, Genetics, Maternal and Child Health, University of Genoa, 16147 Genoa, Italy; U.O.C. Genetica Medica, IRCCS Istituto Giannina Gaslini, 16147 Genoa, Italy; Center for Advanced Biotechnology and Medicine, and Departments of Pharmacology and Medicine, Robert Wood Johnson Medical School, Rutgers the State University of New Jersey, Piscatway, NJ 08854, USA; Centre de Recherche du Centre Hospitalier de l’Université de Montréal (CRCHUM), and Department of Neuroscience, Université de Montréal, Montreal H2X 0A9, Quebec, Canada; Section Clinical Genetics, Department of Human Genetics and Amsterdam Reproduction and Development, Amsterdam University Medical Centers, 1105 AZ, Amsterdam, The Netherlands; Department of Medical Genetics, Division of Child and Adolescent Health, University Hospital of North Norway, 9019 Tromsø, Norway; Medical Molecular Genetics Department, Human Genetics and Genome Research Institute, National Research Centre, Cairo 12622, Egypt; Department of Pediatric Neurology, Children Hospital, University of Child Health Sciences, Lahore, Punjab 54000, Pakistan; Department of Medical Genetics, Oslo University Hospital, 0450 Oslo, Norway; Department of Medical Genetics, Division of Child and Adolescent Health, University Hospital of North Norway, 9019 Tromsø, Norway; Institute of Human Genetics and Institute for Auditory Neuroscience and InnerEarLab, University Medical Center Göttingen, 37073 Göttingen, Germany; Department of Neuromuscular Disorders, UCL Queen Square Institute of Neurology, University College London, London WC1N 3BG, UK; Department of Paediatric Neurology, Children’s Hospital and Institute of Child Health, Multan, Punjab 60000, Pakistan; Department of Translational Genomics, Center for Genomic Medicine, King Faisal Specialist Hospital and Research Center, Riyadh 12713, Saudi Arabia; Clinical Genetics Department, Human Genetics and Genome Research Institute, National Research Centre, Cairo 12622, Egypt; Neuroradiology Unit, IRCCS Istituto Giannina Gaslini, 16147 Genova, Italy; Center for Advanced Biotechnology and Medicine, and Departments of Pharmacology and Medicine, Robert Wood Johnson Medical School, Rutgers the State University of New Jersey, Piscatway, NJ 08854, USA; Department of Cell Biology and Physiology, and Center for the Investigation of Membrane Excitability Diseases (CIMED), Washington University, St Louis, MO 63110, USA; Division of Habilitation for Children, Innlandet Hospital Sanderud, Hamar 2312, Norway; South East Scotland Genetic Service, Western General Hospital, Edinburgh EH4 2XU, UK; West of Scotland Clinical Genetics Service, Queen Elizabeth University Hospital, Glasgow G51 4TF, UK; Section Clinical Genetics, Department of Human Genetics and Amsterdam Reproduction and Development, Amsterdam University Medical Centers, 1105 AZ, Amsterdam, The Netherlands; Department of Genetics, University Medical Center, Utrecht, 3584 CX, The Netherlands; U.O.C. Genetica Medica, IRCCS Istituto Giannina Gaslini, 16147 Genoa, Italy; Department of Neuromuscular Disorders, UCL Queen Square Institute of Neurology, University College London, London WC1N 3BG, UK; Centre de Recherche du Centre Hospitalier de l’Université de Montréal (CRCHUM), and Department of Neuroscience, Université de Montréal, Montreal H2X 0A9, Quebec, Canada; Department of Cell Biology and Physiology, and Center for the Investigation of Membrane Excitability Diseases (CIMED), Washington University, St Louis, MO 63110, USA; Department of Pediatric Rehabilitation, University Hospital of North Norway, 9019 Tromsø, Norway; Institute of Clinical Medicine, UiT The Arctic University of Norway, 9019, Tromsø, Norway; Center for Advanced Biotechnology and Medicine, and Departments of Pharmacology and Medicine, Robert Wood Johnson Medical School, Rutgers the State University of New Jersey, Piscatway, NJ 08854, USA

**Keywords:** KATP channels, SUR2, ABCC9, neurodevelopmental disorder

## Abstract

Loss-of-function mutation of *ABCC9*, the gene encoding the SUR2 subunit of ATP sensitive-potassium (K_ATP_) channels, was recently associated with autosomal recessive *ABCC9*-related intellectual disability and myopathy syndrome (AIMS). Here we identify nine additional subjects, from seven unrelated families, harbouring different homozygous loss-of-function variants in *ABCC9* and presenting with a conserved range of clinical features.

All variants are predicted to result in severe truncations or in-frame deletions within SUR2, leading to the generation of non-functional SUR2-dependent K_ATP_ channels. Affected individuals show psychomotor delay and intellectual disability of variable severity, microcephaly, corpus callosum and white matter abnormalities, seizures, spasticity, short stature, muscle fatigability and weakness. Heterozygous parents do not show any conserved clinical pathology but report multiple incidences of intra-uterine fetal death, which were also observed in an eighth family included in this study. *In vivo* studies of abcc9 loss-of-function in zebrafish revealed an exacerbated motor response to pentylenetetrazole, a pro-convulsive drug, consistent with impaired neurodevelopment associated with an increased seizure susceptibility.

Our findings define an *ABCC9* loss-of-function-related phenotype, expanding the genotypic and phenotypic spectrum of AIMS and reveal novel human pathologies arising from K_ATP_ channel dysfunction.

## Teaser

Efthymiou *et al.* characterize nine individuals with *ABCC9*-related intellectual disability and myopathy syndrome (AIMS). All nine individuals have homozygous loss-of-function variants in the *ABCC9* gene, which encodes SUR2, a regulatory subunit of ATP-sensitive potassium channels.

## Introduction

*ABCC9*-related intellectual disability and myopathy syndrome (AIMS; OMIM #619719) was recently identified in six individuals, from two families, who were all homozygous for the same loss-of-function (LoF) splice-site variant in *ABCC9* (NM_005691:c.1320+1G>A).^1^*ABCC9* encodes the SUR2 (sulfonylurea receptor 2) subunit of ATP-sensitive potassium (K_ATP_) channels, which are widely expressed throughout the body. K_ATP_ channels are nucleotide-regulated potassium channels, comprising pore-forming K_ir_6 subunits co-assembled with regulatory SUR subunits, that couple cellular metabolism and diverse cellular signalling pathways to the membrane potential.^2^

The two mammalian K_ir_6 isoforms, K_ir_6.1 (*KCNJ8*) and K_ir_6.2 (*KCNJ11*) and two SUR isoforms, SUR1 (*ABCC8*) and SUR2 (*ABCC9*), show distinct properties and tissue expression patterns. SUR proteins are members of the ABC-transporter family and share core structural features of two transmembrane domains (TMD1 and TMD2) and two nucleotide binding domains (NBD1 and NBD2) with family members such as CFTR (*ABCC7*) and the multidrug resistance protein (MRP-1; *ABCC1*). SURs have no recognized transporter function, but instead, regulate K_ATP_ channel complexes, conferring Mg-nucleotide activation and pharmacological sensitivity and modulating ATP inhibition.^2^ Functional K_ATP_ channel expression at the plasma membrane requires co-assembly of four K_ir_6 subunits with four SUR subunits.^3-9^ Extensive study has shown that truncation of SUR proteins impairs or abolishes surface expression of K_ATP_ channels^10,11^ and truncations of SUR1 are associated with congenital hyperinsulinism due to loss of pancreatic K_ATP_ function.^12^

SUR2-containing K_ATP_ channels are well described in multiple tissues, including smooth, cardiac and skeletal muscle.^13,14^ Channel activity serves to hyperpolarize the membrane potential in smooth muscle, reducing vascular tone, gastrointestinal motility and lymphatic contractility.^15-19^ In striated muscle, channel activation results in action potential shortening in cardiac muscle and decreased action potential amplitudes and membrane potential hyperpolarization in skeletal muscle.^20-24^ Additional roles for SUR2-containing channels have been proposed in diverse tissues including the brain, bone, hair follicles, fibroblasts and the endothelia.^13,25-29^

The previously reported AIMS individuals displayed cognitive impairment, muscle weakness, fatigability, facial dysmorphism, white matter hyperintensities and cardiac systolic dysfunction in older individuals. Some of these features, such as the musculoskeletal and cardiac dysfunction, were predicted from earlier studies of K_ATP_ channel gene knockout mice.^20,23,24,30-32^ In contrast, cognitive and neurological impairment remains to be explained.

We now report nine new individuals, from seven unrelated families, harbouring biallelic variants in *ABCC9* who present with a distinctive neurodevelopmental phenotype consistent with previously reported AIMS patients, and associated with imaging features resembling periventricular leukomalacia and brain calcifications. Each family presents with different *ABCC9* variants that are predicted to result in major deletions or truncations of the SUR2 protein, and which we show lead to complete LoF of recombinant K_ATP_ channels. Novel genotypes associated with phenotypes that are consistent with previously reported AIMS patients, and the identification of additional novel features, expands this *ABCC9* LoF-associated recessive disorder.

## Materials and methods

### Patients

Written informed consent was obtained from the parents or legal guardians of all enrolled individuals. Patient data were anonymized before sharing. Subjects were recruited from several clinical and research centres in Europe, Africa, the Middle East and Asia (Department of Human Genetics, Amsterdam University Medical Center, Amsterdam, The Netherlands; University Hospital of North Norway, Tromsø, Norway; UCL Queen Square Institute of Neurology, UK; National Research Centre, Cairo, Egypt; King Abdullah International Medical Research Center, Riyadh, Saudi Arabia; Multan Children’s Hospital, Multan, Pakistan; King Faisal Specialist Hospital and Research Center, Riyadh, Saudi Arabia; and West of Scotland Clinical Genetics Service, UK).

### Clinical evaluation

Developmental history, behavioural disturbances, neurological examinations and electro-clinical findings were collected from clinical charts and thoroughly reviewed by the referring physicians and paediatricians with expertise in paediatric neurology. Brain MRIs were performed locally and neuroimaging findings were systematically reviewed by an expert paediatric neuroradiologist. Molecular and clinical findings of previously reported AIMS patients^1^ were reviewed and compared with the current cohort.

### Genotyping

Whole exome sequencing was performed, where indicated, on genomic DNA extracted from peripheral blood leucocytes separately at three different laboratories, as previously described.^33^ Genetic variants were filtered according to allele frequency ≤0.001 in the Genome Aggregation Database (gnomAD; https://gnomad.broadinstitute.org), presence in ClinVar (https://www.ncbi.nlm.nih.gov/clinvar/), conservation [Genomic Evolutionary Rate Profiling (GERP) http://mendel.stanford.edu/SidowLab/downloads/gerp/] and predicted impact on protein structure and function. The pathogenicity of candidate variants was predicted using Combined Annotation Dependent Depletion (CADD, GRCh37-v1.6 version, https://cadd.gs.washington.edu), Sorting Intolerant From Tolerant (SIFT, https://sift.bii.a-star.edu.sg) and PolyPhen-2 (http://genetics.bwh.harvard.edu/pph2/). Whole genome sequencing was performed, where indicated, on genomic DNA extracted from peripheral blood leucocytes with trio filtration of variants in an exome panel containing 18 678 genes. Genetic variants were filtered according to allele frequency ≤0.005 in gnomAD for genes associated with autosomal dominant disorders and ≤0.01 for remaining variants. American College of Medical Genetics and Genomics and the Association for Molecular Pathology (ACMG/AMP) guidelines were used to classify candidate variants.^34^ Sanger sequencing was performed to validate the detected variants and for segregation analysis. *ABCC9* variants are reported according to RefSeq NM_005691 (GenBank NC_000012.12), using HGVS recommendations.^35^ The variants were submitted to the Leiden Open Variation Database (LOVD, https://www.lovd.nl) with the following accession numbers: #00428407, #00428408, #00428409, #00428410, #00435232, #00435233 and #00435234.

### Minigene splicing assay

RNA studies assaying effects of the c.284+1G>A and c.4212-1G>T canonical splice variants were performed, as previously described.^36,37^ Coding exons 2 (142 bp) and 35 (104 bp), with flanking intronic sequences, were directly PCR amplified from a control individual and each proband with primers containing additional restriction sites (for exons 2 and 35, respectively: forward primers with a XhoI restriction site: 5′-aattctcgagCCATGTTGTCATCCAGAGTTG-3′ and 5′-aattctcgagTGGCAGCACAGCTGATCTAA-3′ and reverse primers with a BamHI restriction site: 5′-attggatccCAACAAACCTCCGTGACTCAA-3′ and 5′-attggatccCAATGACCTGTACCCACCAA-3′). PCR fragments were ligated into the pSPL3 exon trapping vector between exon A and exon B and confirmed by Sanger sequencing.

Vectors containing the *ABCC9* c.284+1G>A or c.4212-1G>T variants or wild-type sequences were transfected into HEK 293 T cells (ATCC). An empty vector and transfection negative reactions were included as controls. Transfected cells were harvested 24 h after transfection. Total RNA was extracted using miRNeasy Mini Kit (Qiagen) and reverse transcribed using a High-Capacity RNA-to-cDNA Kit (Applied Biosystems). cDNA was PCR amplified using forward (5′-TCTGAGTCACCTGGACAACC-3′) and reverse (5′-ATCTCAGTGGTATTTGTGAGC-3′) primers. Amplified fragments were visualized by gel electrophoresis and Sanger sequenced.

### Recombinant K_ATP_ channel studies

Human SUR2A (accession no. NM_005691) encoding sequences were synthesized and cloned into pcDNA3.1(−) using NheI and XhoI endonucleases. HEK293 cells (Millipore Sigma) were transfected (Fugene 6, Promega) with wild-type pcDNA3.1_mKir6.2 (GenBankTM accession no. D50581.1) and wild-type or mutant hSUR2A constructs in addition to pcDNA3.1_eGFP for visual detection of transfection. Cells transfected with pcDNA3.1-eGFP alone were used as a negative control.

**Figure 1:**
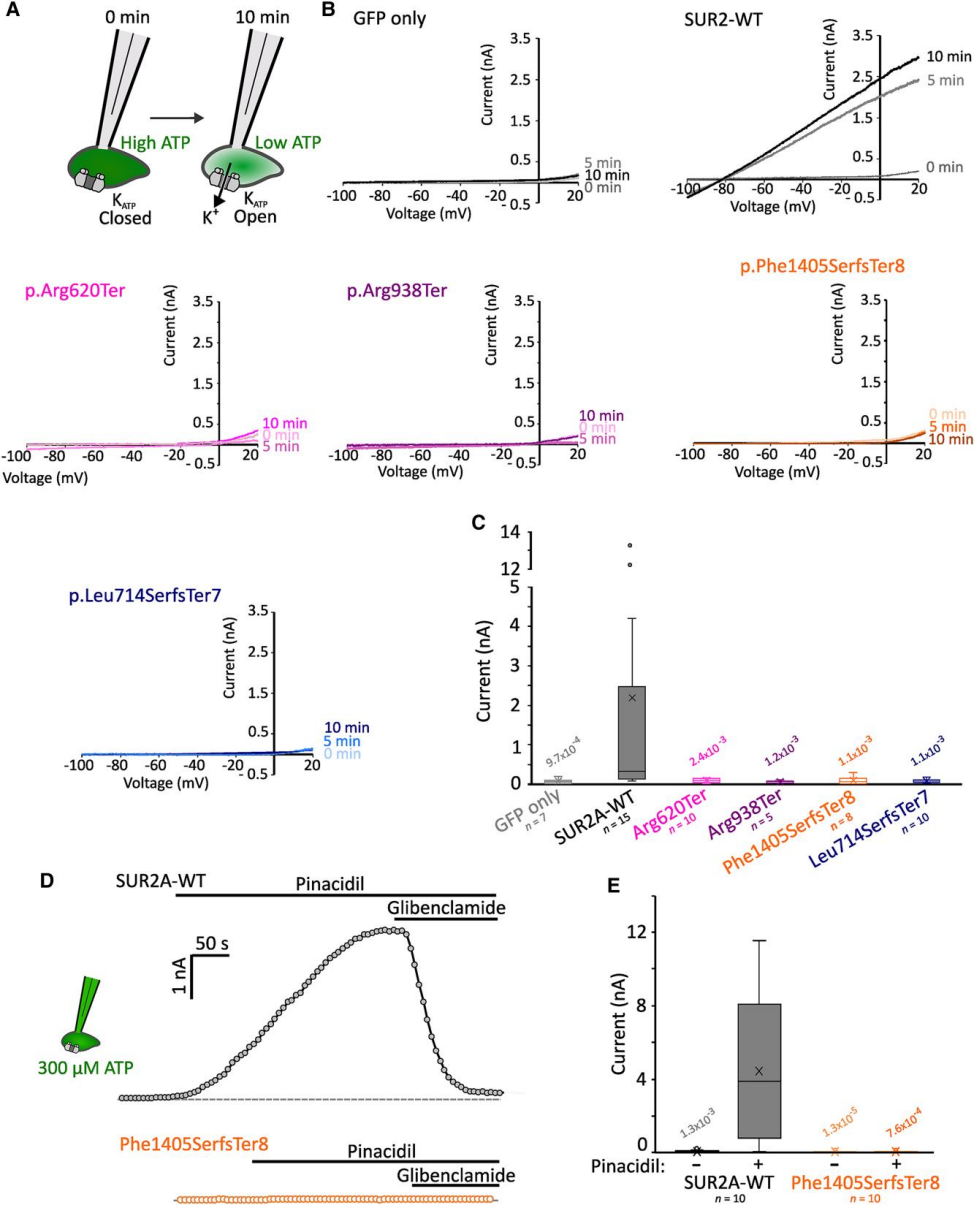
**AIMS associated mutations cause complete K_ATP_ channel loss-of-function**. (**A**) Whole-cell patch clamp recordings were performed in HEK293 cells transfected with K_ir_6.2 and wild-type (WT) or mutant SUR2A. Initial ambient levels of intracellular ATP means channels are inhibited immediately after membrane rupture to whole-cell configuration. Over time, ATP levels are depleted by dilution with the pipette solution. (**B**) Example current traces from voltage ramps for cells transfected with GFP alone, or K_ir_6.2 alongside SUR2A-WT, SUR2[Arg620Ter], SUR2[Arg938Ter], SUR2[Phe1405SerfsTer8] or SUR2[Leu714SerfsTer7]. (**C**) Summary showing whole-cell currents at 0 mV measured at 10 min after establishing the whole-cell recording configuration. Box and whisker plot shows median as horizontal line, mean as ‘X’, and interquartile range as coloured box. *P*-values from Dunn’s pairwise comparisons versus SUR2A-WT following Kruskal-Wallis test shown. (**D**) K_ATP_ currents were recorded from cells transfected with K_ir_6.2 and SUR2A-WT (*top*, grey) or SUR2[Phe1405SerfsTer8] (*bottom*, orange). Whole cell currents were recorded from ramp protocols as shown above with 300 µM ATP included in the patch pipette. Currents at 0 mV from sweeps recorded at 5-s intervals are shown. K_ATP_ channels from SUR2A-WT expressing cells displayed robust activation upon administration of 100 µM pinacidil, which was reversed by the K_ATP_ inhibitor glibenclamide (10 µM). Dotted line shows zero current level. (**E**) Summary of currents recorded prior to and after pinacidil administration in cells transfected with K_ir_6.2 and SUR2A-WT or SUR2[Phe1405SerfsTer8]. *P*-values from Dunn’s pairwise comparisons versus SUR2A-WT currents in pinacidil following Kruskal-Wallis test shown. GFP = green fluorescent protein.

Patch clamp recordings were made from cells 36–48 h post-transfection using an Multiclamp 700B amplifier and Digidata 1550B digitizer (Molecular Devices). Currents recorded in response to voltage ramps from −100 to +60 mV from a holding potential of −80 mV were sampled at 10 kHz, low-pass filtered at 1 kHz. The bath solution contained (in mM): 136 NaCl, 6 KCl, 2 CaCl_2_, 1 MgCl_2_, 10 HEPES, 10 glucose (pH 7.4 with NaOH). The pipette solution without ATP contained (in mM): 140 KCl, 10 NaCl, 1 MgCl_2_, 10 HEPES, 0.5 CaCl_2_, 4 K_2_PHO_4_ and 5 EGTA (pH 7.3 with KOH). To test pinacidil activation, 300 µM ATP (potassium salt) was added to the pipette solution and pinacidil (100 µM) and glibenclamide (10 µM) were administered during recordings. Glass micropipettes were pulled from thin-wall borosilicate glass (Sutter) with resistances of 2.5–4 MΩ when filled with pipette solution. Recordings were performed at 20°C–22°C. Whole-cell currents were measured immediately after membrane rupture and for 10 min thereafter. K_ATP_ currents increased over time in wild-type SUR2A expressing cells as intracellular ATP was diluted by the pipette solution (Fig. 1A). Leak-subtraction was applied by measuring conductances at −80 mV (the theoretical reversal potential for potassium in these conditions) and currents at 0 mV after 10 min are reported. Patch clamp data were analysed with a Kruskal-Wallis omnibus test followed by Dunn’s tests for pairwise comparisons.

### Zebrafish model

#### Zebrafish development and maintenance

Zebrafish (*D*a*nio rerio*) carrying a 13-base frame-shift deletion (XM_005164706.4 c.2947_2959del) in the *abcc9* gene were initially generated by CRISPR/Cas9 mutation of Tübingen longfin one-cell-stage embryos, as previously described.^1,38^ This deletion results in reduced expression of *abcc9* transcripts,^1^ is predicted to result in truncation of any translated protein (XP_005164763.1 p.Gly983TrpfsTer4) and has been shown to result in a loss of SUR2-dependent K_ATP_ channel expression.^39^ Zebrafish were maintained as homozygous SUR2-STOP mutants, which were crossed to generate larvae used in locomotor assays. SUR2-STOP larvae were compared with larvae from in-crossed Tübingen longfin wild-type controls.

#### Larval locomotor assay

We monitored the swimming behaviour of 7 days post-fertilization (dpf) larvae separated into single wells of a 96-well plate containing 200 µl of E3 media and habituated in the Daniovision (Noldus) recording chamber for 1 h before the start of the experiment. Swimming was monitored over a baseline 1-h dark period in the absence of followed by a 1-h dark period after 3 mM pentylenotetrazol (PTZ, Sigma-Millipore) administration. Ethovision XT12 (Noldus) was used to analyse distances swam.

#### Morphological analysis

Morphological analysis was performed on 7 dpf animals. Larvae were immobilized in a 3% methylcellulose cavity and images were taken using a stereomicroscope (Leica S6E). Body length, head and eye sizes were measured from scale-calibrated images using ImageJ (National Institutes of Health, Bethesda, Maryland).

## Results

### Patient descriptions

Nine novel AIMS subjects with homozygous *ABCC9* variants were identified in seven unrelated families exhibiting clinical features that overlap with those previously observed^1^ (Tables 1 and 2).

**Table 1:** Demographic, genetic and key clinical features of AIMS patients

Patient ID	1-1	2-1	2-2	3-1	4-1	5-1	6-1	6-2	7-1
Family ID	F1	F2	F2	F3	F4	F5	F6	F6	F7
**Demographics**									
Age	31 years	7.5 years	10.5 years	4.5 years	14 years	4 years	13 years	10 years	36 years
Sex	F	F	F	F	F	F	M	F	F
Nationality	Norwegian	Pakistani	Pakistani	Egyptian	Dutch	Egyptian	Norwegian	Norwegian	British Pakistani
**Genetics**									
gDNA (hg38)	chr12-21910156-C-T	chr12-21848204-G-A	chr12-21848204-G-A	chr12-21809956-C-A	chr12-21887879-G-A	chr12-21910243-G-A	chr12-21910243-G-A	chr12-21910243-G-A	chr12-21818174del
cDNA^a^	c.1320+1G>A	c.2812C>T	c.2812C>T	c.4212-1G>T	c.1858C>T	c.1234C>T	c.284+1G>A	c.284+1G>A	c.3747del
Protein	p.(Ala389_Gln440del)	p.(Arg938Ter)	p.(Arg938Ter)	p.(Phe1405SerfsTer8)	p.(Arg620Ter)	p.(Gln412Ter)	p.(Phe49GlyfsTer13)	p.(Phe49GlyfsTer13)	p.(Leu1250TrpfsTer9)
Consanguinity	No	Yes	Yes	Yes	No	Yes	No	No	Yes
**Select clinical features**									
Developmental delay	Global	Global	Global	Motor	Global, mild	Global	Mild DD	Mild DD	Global
Intellectual disability	Severe	Severe	Severe	No	Mild	Mild	Mild	Learning difficulties	Mild learning disability
Microcephaly	No	Yes	Yes	Yes	No	Yes	Yes	No	Yes
Spasticity	Yes	Yes	Yes	Yes	No	Yes	Yes	Yes	No
Seizures	Yes	Yes	No	No	Yes	No	Yes	No	No
Fatigability	Yes	NA	NA	Yes	Yes	Yes	Yes	Yes	Yes
White matter signal alterations	Yes	No	No	Yes	No	Yes	Yes	Yes	NA

DD = developmental delay; F = female; M = male; NA = not assessed.

^a^NM_005691.

**Table 2:** Summary of the cardinal clinical features in AIMS patients

Clinical features	Our cohort (*n* = 9)	%	Previous AIMS patients^1^ (*n* = 6)	%	Total (*n* = 15)	%
Developmental delay	9	100	6	100	15	100
Intellectual disability	8	89	6	100	14	93
Fatigability	7	78	6	100	13	87
White matter signal alterations	5	56	6	100	11	73
Lordosis/scoliosis	5	56	5	83	10	67
Dysmorphism	4	44	6	100	10	67
Neuropsychiatric manifestations	5	56	4	67	9	60
Contractures	5	56	4	67	9	60
Microcephaly	6	67	2	33	8	53
Corpus callosum hypoplasia/agenesis	6	67	0	0	6	40
Seizures	4	44	1	17	5	33
Cardiac abnormalities	1	11	2	33	3	20
Other MRI abnormalities	2	22	0	0	2	13

#### Family 1

**Figure 2:**
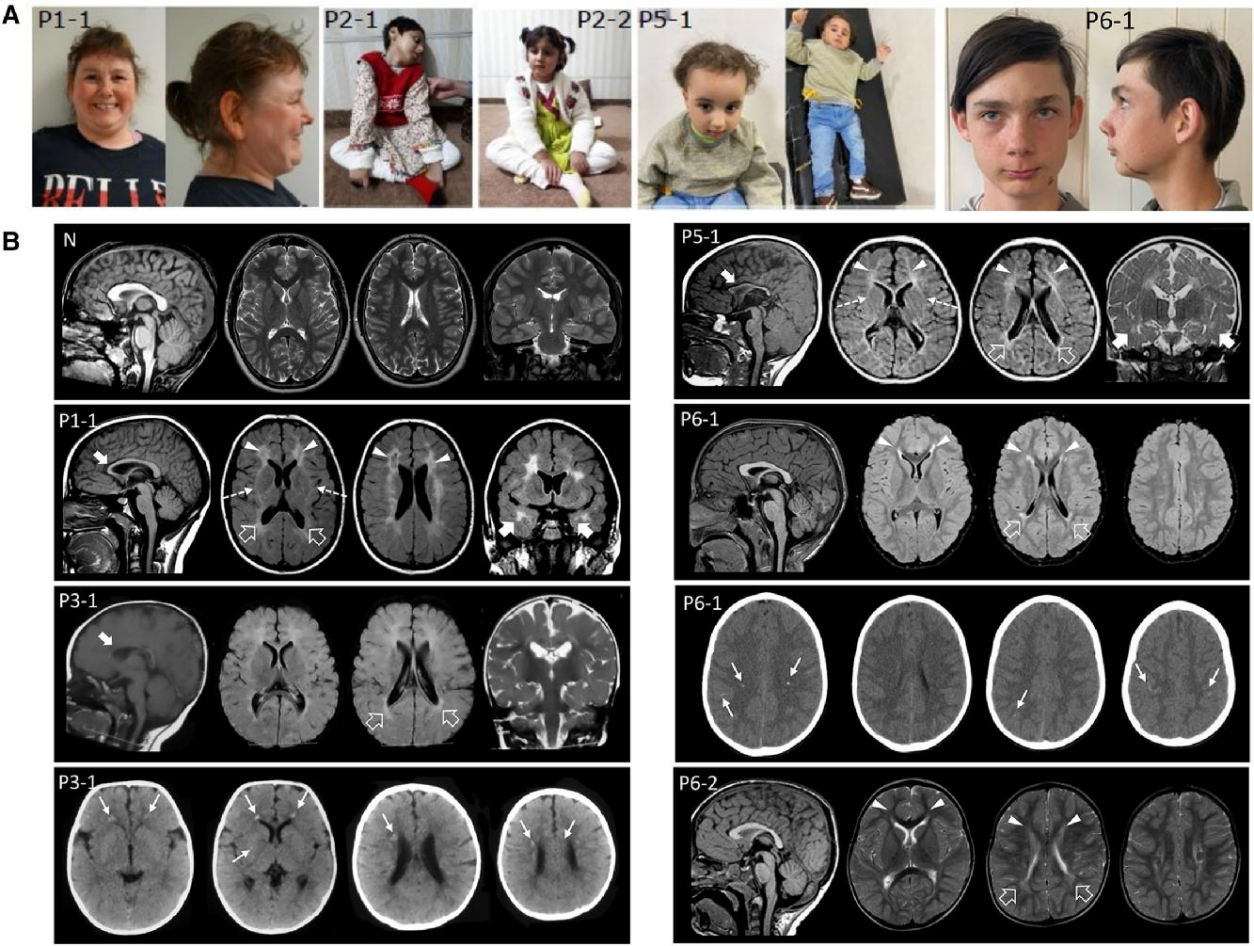
**Clinical features and neuroradiological phenotype of *ABCC9* patients**. (**A**) Clinical photographs. Patient 1-1 at age 30 exhibits hypotelorism, broad nasal tip and large frontal incisors. Patients 2-1 and 2-2 show a variable association of cognitive impairment and spasticity, with a more severe involvement and decerebrate posture in Patient 2-1. Patient 5-1 shows microcephaly, hypotonia, spasticity, drooling and kyphosis. She also has dysmorphic features consisting of bossing forehead, sparse thin hair, epicanthic folds, prominent nose, retrognathia and low set ears. Patient 6-1 at age 13 shows synophrys, anteverted nostrils, thin upper lip and small chin. Epidermal scar-like nevus left cheek. (**B**) Neuroimaging findings of patients compared with a normal control. Brain MRI studies with sagittal T_1_-weighted (*far left image*), axial T_2_ or fluid-attenuated inversion recovery (FLAIR) (*middle two images*) and coronal T_2_ or FLAIR images (*far right image*) performed in Patient 1-1 at 15 years of age, Patient 3-1 at 9 months of age, Patient 5-1 at 10 months of age, Patient 6-1 at 7 years of age, and Patient 6-2 at 1 years and 8 months of age. Head CT, axial images, performed in Patients 3-1 and 6-1 at 6 months and 7 years, respectively. There is reduction of parieto-occipital white matter volume with T_2_/FLAIR hyperintensities and squared-appearance of the lateral ventricles in all subjects (empty arrows). The signal abnormalities extend to the frontal lobes in Patients 1-1, 5-1, 6-1 and 6-2 (arrowheads) and to the anterior temporal regions in Patients 1-1 and 5-1 (thick arrows). Note the small cavitations in the frontal regions in Patient 1-1 and the involvement of the anterior portions of the external capsules (dashed arrows) in Patients 1-1 and 5-1. The corpus callosum is thin in Patients 1-1, 3-1 and 5-1 (curved arrows). Axial CT images reveal multiple small calcifications at the level of the frontal periventricular white matter and right putamen in Patient 3-1 and at the level of the fronto-parietal white matter and cortex in Patient 6-1 (thin arrows).

Patient 1-1 (Fig. 2) is the only daughter born to non-consanguineous healthy parents of Norwegian ancestry. She was delivered in pregnancy Week 38 by caesarean section due to bleeding after an otherwise normal pregnancy. Except for being small for gestational age, the neonatal course was uneventful.

The patient had hypotonia and delayed psychomotor development (Table 1). At the age of 6 weeks, she suffered generalized tonic-clonic seizures (GTCS) (Supplementary Table 1), but to date, has never had seizures again. Neuropsychological examination revealed hypotonia, left-sided hemiplegia, spasticity and anxious behaviour. She was overweight and showed lumbar lordosis, bilateral Achilles tendon contractures and dysmorphic facial features (hypotelorism, broad nasal tip, large upper incisors). Physical examination revealed a small head and short stature.

At the age of 1 she experienced a first episode of coma followed by several similar unexplained episodes during childhood. At the age of 2, an episode of coma/somnolence lasting 1 week was followed by transient left-sided hemiparesis. During that episode, creatine kinase (CK) was 1030 U/l initially, but increased to 36 000 U/l, leading to diagnosis of rhabdomyolysis. She experienced further similar episodes, some with coma and a slight rise in CK, and some with substantial rhabdomyolysis. A muscle biopsy performed after these episodes showed pathological mitochondria and ragged red muscle fibres, but a later biopsy was deemed normal. No further episodes have occurred in the last 5–6 years. She developed left-sided spastic cerebral palsy after the comatose episodes. The patient complained of easy fatigability with cramping within short walking distances. Gene sequencing panels for neuromuscular disease and known causes of rhabdomyolysis have been negative. Her father has experienced one unexplained episode of compartment syndrome and rhabdomyolysis (Supplementary Table 2).

At the age of 1.5 years and 15 years, brain MRI showed mild reduction of white matter volume with squared lateral ventricles, multiple confluent fronto-temporo-parietal signal alterations and periventricular cavitations in the frontal regions resembling severe periventricular leukomalacia (Fig. 2). Also observed were thinning of the anterior portion of the corpus callosum and multiple dilated perivascular spaces at the level of the basal ganglia (Fig. 2) and slightly smaller volume of the hippocampi (Supplementary Fig. 1). Brain MRI spectroscopy performed at the level of the affected white matter and right basal ganglia showed normal spectra. Neuropsychological evaluation in adulthood showed severe intellectual disability. She also has anxiety and episodes of psychosis, for which she is currently medicated. She lives in a sheltered home, now aged 31. Cardiac ultrasound was normal at ages 16 and 31 and a recent 24 h ECG recording displayed no arrythmia.

Trio whole genome sequencing identified the previously reported^1^ variant in *ABCC9*(NM_005691):c.1320+1G>A, p.(Ala389_Gln440del). The variant was homozygous in the affected proband and heterozygous in both parents. No additional disease-causing variants were identified.

#### Family 2

Patients 2-1 and 2-2 (Fig. 2) are affected siblings born to consanguineous healthy parents of Pakistani ancestry. Pregnancy and neonatal course were uneventful, but Patient 2-1 was born prematurely and delivered via caesarean section. Both patients were diagnosed with a global impairment of psychomotor development in the first year of life with delayed milestones, delayed speech development and no response to stimuli (Table 1 and Supplementary Table 1). Patient 2-1 suffered GTCS at the age of 15 months. EEG revealed multifocal interictal epileptic discharges, predominant over right hemisphere. She has been administered valproic acid and levetiracetam. Neurological examination of both siblings revealed microcephaly, severe cognitive dysfunction, decerebrate posture, spasticity, brisk reflexes and drooling. Patient 2-1 receives gastrostomy feeding. Auditory brainstem response and audiometry studies were normal. Ophthalmologic evaluation showed bilateral optic disc pallor. Brain MRI at the age of 3 years old showed partial agenesis of the corpus callosum in both children, which was associated with bilateral polymicrogyria and enlarged CSF spaces in Patient 2-2.

Exome sequencing revealed a homozygous (NM_005691):c.2812C>T, p.(Arg938Ter) variant in the affected siblings. Both parents were heterozygous carriers.

#### Family 3

Patient 3-1 is the affected daughter of consanguineous healthy parents of Egyptian ancestry. Pregnancy and neonatal course were uneventful. Physical examination during infancy revealed psychomotor delay and hypotonia (Table 1 and Supplementary Table 1). At the age of 4, neurological examination revealed microcephaly, spasticity, nystagmus and anxious behaviour. Mild hip girdle weakness and contractures were present, requiring tenotomy. Brain CT performed at 6 months of age showed multiple small calcifications in the periventricular frontal white matter and right basal ganglia. Brain MRI at the age of 9 months showed mild volume reduction of the periventricular white matter with squared appearance of the lateral ventricles, multiple confluent periventricular white matter signal alterations in the fronto-temporo-parietal regions and a thin anterior corpus callosum resembling periventricular leukomalacia (Fig. 2).

Exome sequencing revealed the homozygous (NM_005691):c.4212-1G>T variant in the affected proband, both parents were heterozygous carriers.

#### Family 4

Patient 4-1 is the affected daughter of non-consanguineous healthy parents of Dutch ancestry. After an uncomplicated pregnancy the neonatal course was complicated by hypotonia and unilateral hip dysplasia at birth, for which tendon release was performed at the age of 18 months, lower extremity asymmetry, scoliosis and lordosis. Feeding problems were reported but growth parameters were within normal range. At 11 years of age, she was diagnosed with psychomotor delay (Supplementary Table 1). Neurological examination identified mild cognitive impairment. Brain MRI at the age of 13 years revealed no abnormalities.

Exome sequencing revealed a homozygous (NM_005691):c.1858C>T, p.(Arg620Ter) variant in the affected proband, carried by both heterozygous parents. The family also suffered two intra-uterine fetal deaths (IUFDs) (at 31 weeks and 38 weeks of gestation). Both fetuses were homozygous for the c.1858C>T, p.(Arg620Ter) variant. Two healthy brothers did not carry the variant.

#### Family 5

Patient 5-1 is the daughter of consanguineous healthy parents of Egyptian ancestry, born by caesarean section following an uncomplicated pregnancy. She exhibits defective balance, fatigability and hypotonia, spasticity, weakness and hyperreflexia of the lower limbs. Brain MRI at 10 months of age showed moderate periventricular white matter volume reduction with squared appearance of the lateral ventricles and multiple confluent periventricular white matter signal alterations in the fronto-temporo-parietal regions, resembling a severe periventricular leukomalacia (Fig. 2). A small pons and thin corpus callosum were also noted. Brain CT at 2 years of age showed no calcifications.

Exome sequencing revealed the presence of the (NM_005691):c.1234C>T, p.(Gln412Ter) variant in the homozygous state for the affected proband, carried by both heterozygous parents.

#### Family 6

Patient 6-1 (Fig. 2) is the second child of healthy, unrelated Norwegian parents. He was born prematurely at 33 + 6 weeks of gestation. Apgar score was 5-8-9 (at 1, 5 and 10 min) and he was resuscitated. Delayed psychomotor development became evident and he was diagnosed with unilateral cerebral palsy. Brain MRI at 7 years of age displayed mild reduction of periventricular white matter in the occipito-parietal regions with squared appearance of the lateral ventricles. There were multiple confluent fronto-parietal signal alterations, mainly in the periventricular regions, resembling periventricular leukomalacia. Brain CT performed at the same age showed multiple small calcifications in the deep fronto-parietal white matter, and faint linear cortical calcifications in the perirolandic regions (Fig. 2) as well as bilateral calcifications in grey matter. Achilles tendon contracture was treated with botulinum toxin and Achilles tendon lengthening was performed at 7 years of age. He is easily tired when exercising. He is microcephalic, with growth otherwise in the normal range. He was diagnosed with mild intellectual disability by formal neuropsychological testing, has anxiety and takes medication for attention deficit disorder (Supplementary Table 1). At the age of 13, he experienced GTCS, with electric activity suspicious for epilepsy in a sleep-deprived EEG.

Patient 6-2 is the third child in the family and the sister of Patient 6-1. Her mother had premature rupture of the membranes in gestational week 32. She was born at 37 weeks, with an uneventful neonatal course. Global developmental delay was evident. A diagnosis of bilateral spastic cerebral palsy was given at age 1.5 years and brain MRI showed very mild white matter volume reduction and fronto-parietal white matter signal changes resembling periventricular leukomalacia. Achilles tendon contractures were treated with botulinum toxin injections and at 6 years of age gastrocnemius release and tendon lengthening were performed. Motor fatigability is evident. At 9 years of age she had a formal neurocognitive test and was diagnosed with learning difficulties—with skills in the lower normal range. Genome sequencing of both affected siblings revealed homozygosity for the (NM_005691):c.284+1G>A variant with the parents being heterozygous carriers.

#### Family 7

Patient 7-1 is a 36-year-old female and one of five children of healthy British Pakistani parents, who are first cousins. She was born at term after an unremarkable pregnancy. She was described as a ‘floppy baby’ and had feeding difficulties. She underwent a patent ductus arteriosus closure procedure around 6 months of age. There were several admissions to hospital with diarrheal illnesses in early life. She was diagnosed with acquired hypothyroidism around age 13 years. Development was globally delayed and she attended a special school for children with additional educational needs from age 5 years (Table 1 and Supplementary Table 1). She has a mild intellectual disability and lives with her mother, requiring prompting with personal care and some supervision or assistance with most activities of daily living. Aside from relative microcephaly, there was no overt craniofacial dysmorphism. She has a slender, long-limbed habitus, with relatively long fingers. Skin of her hands was affected by dermatitis. There is no history of epilepsy and she has never had brain imaging.

Several episodes of acute psychosis characterized by pressured speech and paranoia have been reported. She has received a diagnosis of autism, associated with symptoms of anxiety and mood lability. There is no formal diagnosis of a muscle disorder, but family reports that she complains of fatigue even after just a short walk. Reflexes were normal. She would not comply with formal muscle examination but was able to walk on tiptoes and heels.

Trio-based analysis of the DDG2P gene panel (https://www.ebi.ac.uk/gene2phenotype) from whole exome sequence data revealed the presence of the (NM_005691):c.3747del, p.(Leu1250TrpfsTer9) variant in the homozygous state for the affected proband, carried by both heterozygous parents.

#### Family 8

In addition to the above cases, we also identified a further family from Saudi Arabia in which both parents carried the *ABCC9* c.2140_2141del, p.(Leu714SerfsTer7) variant and who experienced two IUFDs, at 8 months of pregnancy, of unknown aetiology. A further daughter died 20 days after birth following apnoea. Molecular autopsy by proxy^40,41^ was conducted using exome sequencing on parental DNA, who were found to share the carrier status for the (NM_005691):c.2140_2141del, p.(Leu714SerfsTer7) variant.

### *ABCC9* variants

**Figure 3:**
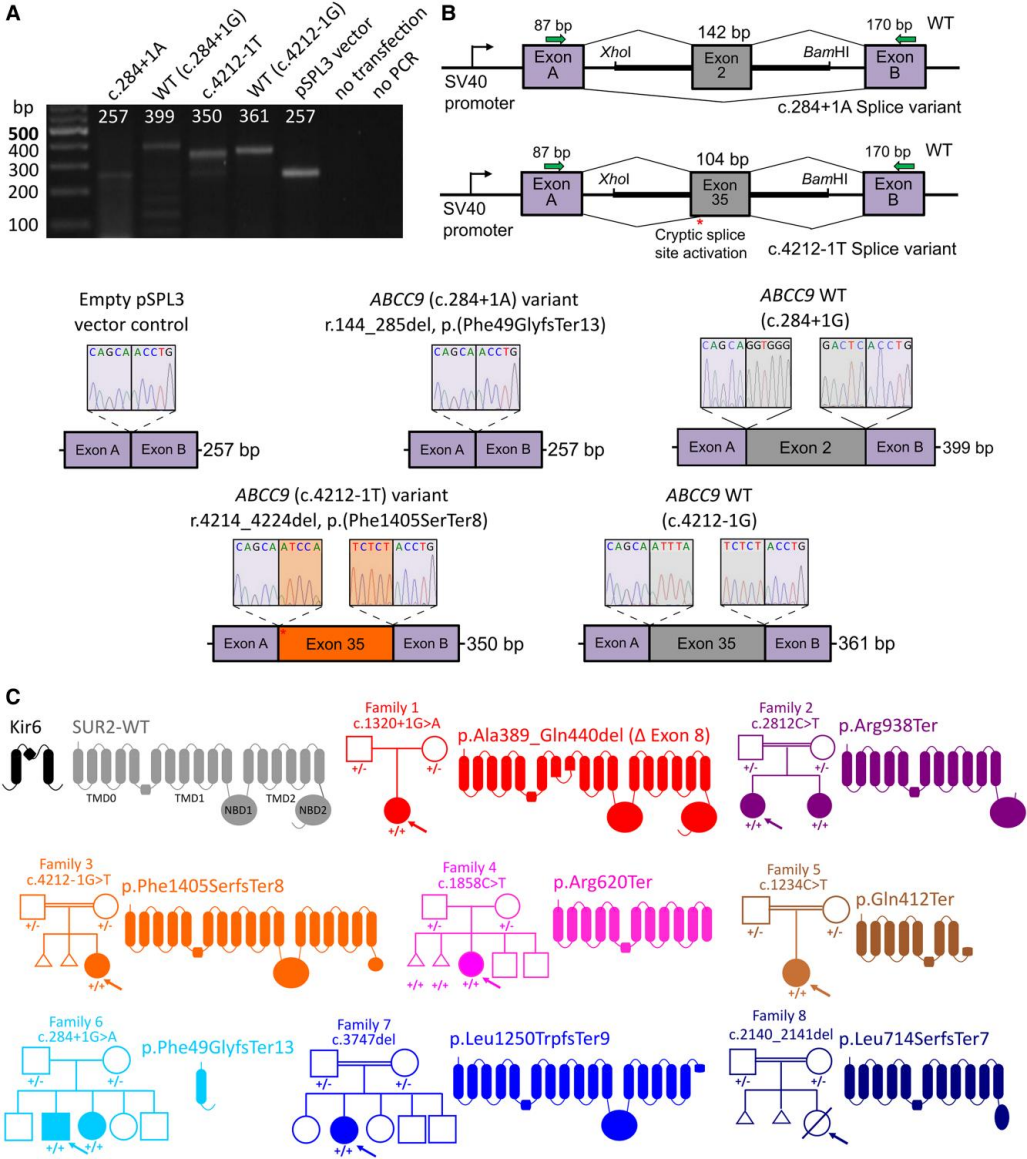
**Molecular consequences of *ABCC9* variants in AIMS individuals**. (**A**) Agarose gel electrophoresis of RT-PCR products showing amplicons from cells transfected with pSPL3 minigene vectors containing either the *ABCC9* c.284+1A or c.4212-1T variant, wild-type (WT) *ABCC9* sequences or the pSPL3 vector alone with no *ABCC9* insertion. (**B**) Schematic representation of the mini-gene construct (*top right*). Exon 2 or 35 of *ABCC9* with the flanking 5′ and 3′ intronic regions was inserted between exon A and exon B of the pSPL3 vector. Sanger sequencing of the RT-PCR amplicons revealed that the c.284+1A variant results in skipping of exon 2 and the c.4212-1T variant resulted in activation of a cryptic splice site resulting in exclusion of 11 bases from exon 37 and the predicted p.(Phe1405SerfsTer8) frameshift. Canonical splicing of wild-type *ABCC9*-containing vector resulted in inclusion of full-length exon 2 or 35. RT-PCR from cells transfected with the empty pSPL3 vector (i.e. no *ABCC9* sequence inserted) resulted in the expected amplification of the pSPL3 exons A and B only. (**C**) K_ATP_ channels assemble as octameric complexes with four K_ir_6 subunits (black) and four SUR subunits (grey). SUR subunits comprise 17 transmembrane domains in three domains (TMD0, TMD1 and TMD2) and two intracellular nucleotide binding domains (NBD1 and NBD2). All variants identified in affected AIMS individuals are predicted or shown to result in splicing defects and major in-frame deletion, or in premature stop codons. Family pedigrees shown for each case; arrow denotes proband.

Exome and genome sequencing identified eight LoF, or predicted LoF, variants in *ABCC9* (Table 1 and Fig. 3). All affected individuals were homozygous for *ABCC9* variants and Sanger sequencing confirmed that unaffected parents were carriers. The variants are rare in the general population (max. allele frequency 0.000088) and absent in homozygous state in the gnomAD database. They are predicted to result either in nonsense-mediated mRNA decay or in the formation of a truncated protein, leading to complete loss of *ABCC9* protein function. All the reported *ABCC9* variants are classified as pathogenic or likely pathogenic according to the ACMG criteria.

The intronic c.4212-1G>T variant identified in Family 3 was assessed in mini-gene splicing studies. Wild-type and mutant *ABCC9* coding exon 35 (notated as exon 37 in Ensembl transcript ENST00000261200.9) and flanking intronic regions were cloned into pSPL3 mini-gene constructs, which were transfected into HEK 293 T cells. Expression of the wild-type intron-exon-intron sequence resulted in the expected canonical splicing (Fig. 3A and B). In contrast, the c.4212-1G>T variant resulted in activation of a cryptic splice acceptor site leading to deletion of 11 nucleotides and a subsequent frameshift (c.4214_4224del, r.4214_4224del, p.(Phe1405SerfsTer8) (Fig. 3A–C). The c.284+1G>A variant resulted in exon skipping of coding exon 2 (142 bp) in the mini-gene assay, which in the native sequence would result in a frame-shift and the p.(Phe49GlyfsTer13) truncation.

### Heterozygous family members

Clinical details from heterozygous parents and relatives are limited but, as shown in Supplementary Table 2, no consistent pathological findings were observed amongst 16 genotyped relatives. Rhabdomyolysis was reported for the carrier father of Patient 1-1, epileptic encephalopathy in the heterozygous maternal aunt of Patient 1-1 and dilated cardiomyopathy was diagnosed at age 60 in the father of Family 2 in the original report of AIMS.^1^ Normal cardiac function was confirmed in three further heterozygous relatives aged >50 years old.

### Effects on K_ATP_ channel function

The novel variants reported here result, or for the c.284+1G>A and c.4212-1G>T variants, are predicted to result, in premature stop codons in *ABCC9* transcripts, which are expected to undergo nonsense-mediated decay *in vivo*. To determine whether any truncated protein, from transcripts which might escape nonsense-mediated decay, would be functional, we co-expressed wild-type or mutant SUR2A with K_ir_6.2 in HEK293 cells (Fig. 1A). Robust potassium conductances were observed in cells expressing wild-type SUR2A in whole-cell recordings after dilution of intracellular nucleotides with a nucleotide-free pipette solution (Fig. 1B). No K_ATP_ channel activity was observed in cells transfected with SUR2[Arg620Ter], SUR2[Arg938Ter], SUR2[Phe1405SerfsTer8] or SUR2[Leu714SerfsTer7] and whole cell currents were essentially identical to cells transfected with GFP alone (Fig. 1B and C). Therefore, as also previously shown for the c.1320+1G>A, p.Ala389_Gln440del variant,^1^ all tested *ABCC9* variants result in a complete loss of recombinant K_ATP_ channel functional expression.

K_ATP_ channel openers (KCOs) are used clinically for hypertension and angina pectoris.^42^ These drugs bind at a common site in SUR2, formed of multiple transmembrane helices from TMD1 and TMD2.^43,44^ All truncations identified here are expected to abolish or disrupt this binding site, with the possible exception of SUR2[Phe1405SerfsTer8], in which the truncation occurs after TMD2. To test if KCO sensitivity was retained in SUR2[Phe1405SerfsTer8] mutant channels, we performed whole-cell patch clamp recordings with 300 µM ATP in the patch pipette before applying pinacidil to activate channels, followed by application of the K_ATP_ inhibitor glibenclamide. Wild-type channels exhibited robust pinacidil and glibenclamide sensitivity as expected, but no channel activity was observed in cells transfected with K_ir_6.2 and SUR2[Phe1405SerfsTer8], even in the presence of pinacidil (Fig. 1D and E). Therefore, KCOs are highly unlikely to be effective for all AIMS mutations identified to date.

### Seizure susceptibility in SUR2 loss-of-function zebrafish

**Figure 4:**
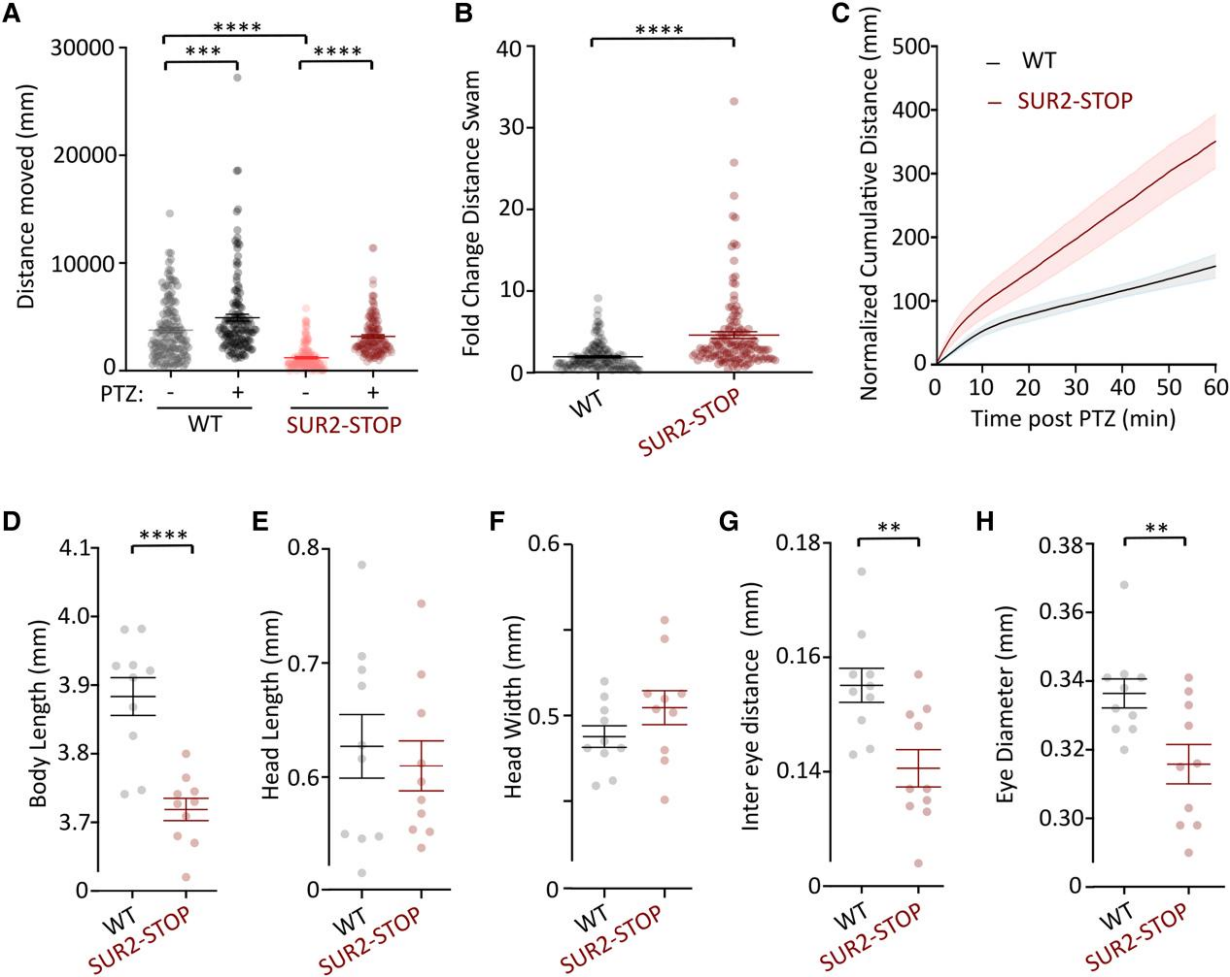
**SUR2-STOP zebrafish larvae exhibit increased seizure susceptibility and dysmorphology**. (**A**) Automated swim tracking was used to measure motility in loss-of-function (LoF) SUR2-STOP fish and wild-type (WT) controls. Swimming distances prior to pentylenetetrazole (PTZ; 3 mM) administration (−) and after PTZ (+) shown. Data from individual measurements from biological replicates as dots with mean and standard error of the mean (SEM) shown. Measurements were made and combined from three separate breeding clutches. ***P < 0.001, ****P < 0.0001 from Tukey tests following one-way ANOVA. (**B**) Swimming distance for each larva after PTZ administration was normalized to basal activity prior to PTZ. Data from individual measurements from biological replicates shown as dots with mean and SEM shown. ****P < 0.0001 according to unpaired *t*-test. (**C**) Plot showing the cumulative distance swam for wild-type and SUR2-STOP larvae after PTZ administration. Swimming distances are normalized to the average swimming distances for each genotype over 30 s prior to PTZ admin. Data shown as mean (solid line) and SEM as shaded bars. (**D**–**H**) Morphometric analysis of wild-type and SUR2-STOP larvae showing reduced body length (**D**), equivalent head dimensions (**E** and **F**) and reduced inter-eye and eye diameter measurements (**G** and **H**) in SUR2-STOP larvae. Data from individual measurements from biological replicates as dots with mean and SEM shown. **P < 0.01 and ****P < 0.0001 according to unpaired *t*-tests.

Seizures were reported in four novel AIMS subjects. Locomotor activity in SUR2-STOP and wild-type zebrafish larvae (7 dpf) was assessed by automated swimming tracking. As previously reported,^1^ SUR2-STOP larvae exhibit reduced basal locomotion, compared to wild-type controls, potentially due to skeletal myopathy (Fig. 4A). Administration of the pro-convulsive GABA receptor antagonist, PTZ (3 mM), to wild-type larvae provoked a small but significant increase in locomotion, reflecting a mild drug-induced hyperactivity. In contrast, locomotion in SUR2-STOP larvae was dramatically increased by PTZ, such that the relative activity of mutant larvae in PTZ, normalized to basal activity, increased to a much greater extent than observed in wild-type larvae (Fig. 4A–C). This suggests abnormal neural excitatory/inhibitory balance in SUR2-STOP larvae, consistent with a neurodevelopmental phenotype and increased seizure susceptibility.

SUR2-STOP larvae also exhibited significantly smaller body length compared to wild-type controls (Fig. 4D), consistent with the small stature observed in the clinical subjects described. SUR2-STOP larvae exhibited facial dysmorphology, with decreased inter-eye distance and eye size, but gross head dimensions were not significantly different (Fig. 4E–H).

## Discussion

We identified seven homozygous *ABCC9* LoF variants in nine affected individuals from seven unrelated families of different ancestry. Seven variants are novel while the c.1320+1G>A (p.Ala389_Gln440) variant of Patient 1-1 is the same detected in the two previously reported Norwegian families. Notably, Family 1 is also from Norway and this variant is enriched in neighbouring northern Finland by two orders of magnitude relative to non-Finnish European populations, suggesting a founder effect [Sequencing Initiative Suomi project (SISu), Institute for Molecular Medicine Finland (FIMM), University of Helsinki, Finland (URL: http://sisuproject.fi) (SISu v4.1, April 2023) 12:22063090; rs139620148]. The p.Ala389_Gln440 deletion arises from the in-frame deletion of exon 8 and results in a complete loss of plasmalemmal K_ATP_ function upon heterologous expression.^1^ The novel variants in *ABCC9* result, or are predicted to result, in premature stop codons and are expected to lead to nonsense-mediated decay and/or major truncation of the SUR2 protein, with deleterious effects on SUR2-dependent K_ATP_ channels. Characterization of recombinant channels demonstrates that p.Arg620Ter, p.Arg938Ter, p.Phe1405SerfsTer8 and p.Leu714SerfsTer7 all result in complete loss of K_ATP_ function. The c.1234C>T, p.(Gln412Ter); c.284+1G>A, p.(Phe49GlyfsTer13); and c.3747del, p.(Leu1250TrpfsTer9) variants were most recently identified and are expected to result in complete LoF.

### Comparison of new patients with initial patients

Previously reported patients belonged to two families in which the same variant (c.1320+1G>A) segregated in six affected members.^1^ These subjects presented with an overlapping spectrum of clinical features, with a core neurological phenotype consisting of psychomotor delay, intellectual disability, anxious behaviour, hyperreflexia and hypotonia in childhood (Table 2). Additionally, they exhibited fatigability and myopathic features. The new cohort showed conserved features, including nystagmus, seizures, tendon abnormalities and lumbar lordosis, but also present with additional distinctive pathologies, expanding the *ABCC9*-related phenotype spectrum (Table 2 and Supplementary Table 1). Neurological examination revealed spasticity and exaggerated deep tendon reflexes in six of nine subjects, with two subjects showing severe decerebrate postures. Progressive microcephaly diagnoses were made for six of nine subjects. A peculiar clinical course was observed in Patient 1-1, who harboured the same variant detected in the first two Norwegian families. This subject exhibited multiple episodes of loss of consciousness, which occurred in combination with rhabdomyolysis. One patient in the original cohort experienced an episode of coma, as well as transient white matter changes on MRI, without significant elevation of CK. Intriguingly, two older female patients experienced episodes of psychosis of unknown mechanisms.

While systolic dysfunction was described in two previous cases,^1^ no significant cardiac disorder was identified in our study. Interestingly, the subjects with cardiac dysfunction were 29 years or older at initial diagnosis, suggesting that a follow-up is important in *ABCC9* patients, as cardiac dysfunction may emerge later in the disease course.

### Comparison of brain MRI findings

Brain abnormalities observed in previous *ABCC9* subjects consisted of white matter signal alterations localized in the centrum semiovale or in the periventricular regions associated with brain calcifications in one case.^34^ Similar findings were observed in Patients 1-1, 3-1, 5-1, 6-1 and 6-2 from our cohort. Of note, we also found brain calcifications in two patients; in the frontal periventricular white matter and right basal ganglia in one subject (Patient 3-1) and in both the white and grey matter in another subject (Patient 6-1). In addition, white matter cavitations were detected in the frontal regions in Patient 1-1.

White matter changes in these patients frequently resemble periventricular leukomalacia (PVL). PVL is often the end-stage of white matter damage in preterm infants, with a likely underlying inflammatory mechanism, however, recent evidence demonstrates that PVL-like features may present in some genetic conditions in the absence of perinatal risk factors. This neuroradiological pattern has been associated with *COL4A1*/*A2*, *AMPD2*, *TBCK* and *NSD1* variants, as well as dehydrogenase deficiency and incontinentia pigmentii.^45-53^ PVL has also been found in subjects with *WWOX*, *SPATA5L1*, *WIPI2* and *EZH2* variants.^54-56^ Therefore, rather than a specific entity, PVL might represent a radiological sign of white matter involvement with specific prenatal timing and mechanisms, that in certain clinical scenarios may help with diagnosis of a genetic condition. Notably, in subjects with developmental delay, intellectual disability and fatigability (Table 2) the presence of PVL-like changes associated with progressive microcephaly, temporal white matter involvement and scattered calcifications should raise the suspicion of an *ABCC9*-related disorder.

We detected additional novel brain abnormalities in some subjects in line with a white matter involvement; hypoplasia or partial agenesis of the corpus callosum was identified in five patients (Patients 1-1, 2-1, 2-2, 3-1 and 5-1). Thinning of the corpus callosum was mainly related to the white matter volume reduction and callosal agenesis is likely related to a primary white matter developmental disorder. Bilateral polymicrogyria was detected in Patient 2-2. These neuroradiological manifestations expand the spectrum of brain MRI abnormalities in *ABCC9* patients, suggesting that white matter is primarily involved in these individuals with a PVL-like pattern, but cerebral malformations may occasionally be part of the spectrum.

### AIMS pathology in a zebrafish model

Consistent dysmorphic features were observed in the original AIMS patients, including hypotelorism, broad nasal tip, flat face and thin upper lip vermillion.^1^ In the current study, facial features were described as normal for five subjects, with the Norwegian individual (Patient 1-1) again exhibiting hypotelorism and broad nasal tip, and one further subject (Patient 5-1) exhibiting other dysmorphology (Table 2 and Supplementary Table 1). LoF SUR2 (SUR2-STOP) zebrafish larvae display reduced intra-orbital distances and eye diameters, linking SUR2 dysfunction with abnormal facial development.

Epilepsy with unconscious episodes was reported in one original AIMS patient.^1^ Here, Patient 1-1 had one unexplained generalized seizure episode in the neonatal period, but none since, and three others (Patients 2-1, 4-1 and 6-1) also experienced seizures as children. Therefore, multiple incidences of epileptic activity have now been observed in unrelated *ABCC9* LoF AIMS patients. To determine whether SUR2 LoF increases seizure susceptibility, we subjected wild-type and SUR2-STOP zebrafish larvae to PTZ-sensitivity tests. SUR2-STOP larvae exhibit reduced basal locomotion, which is likely due to skeletal muscle weakness,^1,57^ but small size or altered vision due to craniofacial dysmorphology in SUR2-STOP fish might also contribute. The epileptogenic GABA-A receptor antagonist PTZ had a markedly greater effect on locomotion in SUR2-STOP larvae than wild-type controls, consistent with increased seizure susceptibility and providing experimental evidence of pro-epileptic effects of SUR2 LoF.

### Intra-uterine fetal death in *ABCC9* variant families

Family 4 suffered two IUFDs of fetuses homozygous for the c.1858C>T, p.(Arg620Ter) variant. In Family 8, who experienced multiple incidences of IUFD, DNA from two fetuses and one neonatal death was unobtainable, but molecular autopsy by proxy showed both parents to be carriers of the c.2140_2141del, p.(Leu714SerfsTer7) variant. In the first report of AIMS,^1^ one family elected to terminate a pregnancy in Week 20 due to severe skeletal dysplasia in the fetus. However, sequencing revealed the affected fetus to be only heterozygous for the c.1320+1G>A variant. Whether IUFD might represent the severe end of the AIMS spectrum is left to determine.

### Pathophysiology and variable severity in AIMS

How *ABCC9* mutations and loss of SUR2-dependent K_ATP_ channel activity result in these diverse neurological and developmental pathologies is not yet fully understood. Neuronal K_ATP_ channels, involved in nutrient and metabolic sensing, have been shown to play neuroprotective roles in ischaemia and protect against hypoxia- and drug-induced epilepsy, where channel activation reduces excitability.^58-61^ SUR1 is reportedly the predominant subunit in most neuronal populations,^62,63^ although SUR2 mRNA has been identified in multiple neurons in rodents, including hippocampal CA1 pyramidal and dentate gyrus granule cells, and dopaminergic excitatory neurons of the substantia nigra pars compacta. K_ATP_ channels in these neurons show an intermediate pharmacological profile that is suggestive of mixed SUR1/SUR2/K_ir_6.2 channel composition.^27-29^ Single-cell mRNA expression databases reveal *ABCC9* expression in excitatory neurons and glial cells^64^ (https://www.proteinatlas.org/ENSG00000069431-ABCC9/single+cell+type). Of note, *ABCC9* polymorphisms have also been associated with hippocampal sclerosis in ageing, though a mechanism is again not yet known.^26,65^ There were no signs of hippocampal sclerosis in the patients studied here, although, Patient 1-1 demonstrated slightly smaller hippocampi without associated T_2_/FLAIR signal alterations and Patient 3-1 had bilateral incomplete hippocampal rotation (Supplementary Fig. 1).

A key role for SUR2-dependent K_ATP_ channels in the cerebral vasculature is also emerging, with smooth muscle or pericyte K_ATP_ activation associated with neurovascular coupling.^66^ Cerebrovascular abnormalities are also seen in Cantú syndrome, including white matter hyperintensities that potentially arise from abnormal cerebral blood flow. Therefore, both overactivity and loss of K_ATP_ channels in the cerebral vasculature might converge in limiting dynamic nutrient and oxygen supply. Twenty-four per cent of Cantú syndrome subjects self-reported history of seizures in a recent study,^67^ but the pathophysiological basis is yet to be determined. Additionally, severe gain-of-function mutations in *KCNJ11* (K_ir_6.2) and *ABCC8* (SUR1) are associated with developmental delay, epilepsy and neonatal diabetes (DEND).^68-71^ Neurodevelopmental abnormalities in AIMS may arise from neuronal and/or non-neuronal mechanisms, such as altered vascular or astrocyte dysfunction. Future studies of tissue-specific *ABCC9* perturbation in animal models may provide insights.

The patients we report here exhibit a spectrum of neurodevelopmental pathology ranging from near normal cognition (Patient 3-1) to severe intellectual disability and decerebrate posture. Recombinant analysis of truncated SUR2 subunits reveal that they are completely non-functional. Interestingly, intellectual disability was mild in five out of six of the previously reported subjects with the c.1320+1G>A splice site mutation. Intellectual disability was moderate in one, and severe in the individual with the same genotype in this cohort. The mildly affected Patient 3-1 here, and Patients 6-1 and 6-2 with mild intellectual disability and only learning difficulties, respectively, also exhibit splice-site mutations. It is tempting to speculate that some transcripts might escape aberrant splicing *in vivo*, and thereby moderate severity, although it is also possible that some patients escape early traumatic consequences or otherwise avoid a developmental threshold effect leading to severe disability.

Fatigability, weakness and cramping were consistently observed in the original AIMS subjects and in these new individuals. SUR2 subunits are critically required for K_ATP_ function in muscle, therefore it is likely that muscle pathology in AIMS arises in a skeletal muscle delimited mechanism.^57^ Studies of K_ir_6.2 and SUR2 knockout mice show that the activation of K_ATP_ channels protects myofibres from sustained depolarization and cytosolic calcium overload,^23,24,31^ and both fatigability and involuntary muscle contraction results from K_ATP_ LoF in skeletal muscle specifically.^57^ It is possible that the episodes of rhabdomyolysis reported here for Patient 1-1, may occur due to muscle breakdown in the absence of the myoprotective effects of K_ATP_ channels. Her otherwise unaffected carrier father also experienced an episode of rhabdomyolysis. No obvious reason has been found by gene sequencing with a neuromuscular panel in the father, or in the whole exome sequencing of Patient 1-1, but it remains possible that other genetic factors may contribute.

## Conclusions

Our studies further confirm the association of LoF variants in *ABCC9* with a neurodevelopmental disorder featuring cognitive impairment, childhood hypotonia, seizures, contractures, spasticity and myopathic features, alongside white matter abnormalities often resembling periventricular leukomalacia. Identification of additional genetically confirmed individuals helps to define the phenotypic spectrum and pathological mechanisms of AIMS, and reveals that some patients are severely affected by neurological involvements and brain abnormalities. *In vivo* studies of *abcc9* LoF in zebrafish show an exacerbated motor response to pentylenetetrazole, consistent with increased seizure susceptibility.

## Supplementary Material

awae010_Supplementary_Data

## Data Availability

All data in this study are available within the article and the Supplementary material.
