# Supplementary material for: Novel loss-of-function variants expand ABCC9-related intellectual disability and myopathy syndrome
Source: Brain. 2024 Jan 13;147(5):1822–36. doi: 10.1093/brain/awae010 (PMC11068106; doi:10.1093/brain/awae010)
Supplement: awae010_Supplementary_Data [file awae010_supplementary_data.pdf]

Supplementary Table 1: Clinical features of AIMS patients.

Patient ID	1-1	2-1	2-2	3-1	4-1	5-1	6-1	6-2	7-1
**Family ID**	F1	F2	F2	F3	F4	F5	F6	F6	F7
**Birth history**									
Pregnancy, birth	CS at week 38 due to fetal distress	Normal	Normal	Normal/CS	Normal	Normal/CS	CS week 33+6 premature labour	PROM week 32, induced delivery week 37	Born at term
Birth weight (SDS)	2.8 kg (-1.8 SD)	2 kg (SD NA)	2.8 kg (SD NA)	3 kg (-0.8 SD)	3.1 kg (-0.03 SD)	2.8 kg (-1.2 SD)	2.6 kg (0.65 SD)	3.2 kg (0.77 SD)	2.7 kg (-1.71 SD)
Birth length (SDS)	45 cm (-2.7 SD)	NA	NA	47 cm (-1.1 SD)	NA	49 cm (-0.2 SD)	44 cm (-1.05 SD)	47 cm (-0.49 SD)	Not available
Birth OFC (SDS)	32 cm (-2.6 SD)	NA	NA	33.5 cm (-0.8 SD)	NA	33 cm (-1.2 SD)	31 cm (-0.65 SD)	34 cm (0.66 SD)	Not available
Hypotonia at birth/childhood	Yes	NA	NA	Yes	Yes	Yes	Yes	No	Yes
**Growth**									
Feeding difficulties	Yes	NA	NA	No	Yes	No	No	No	Yes
Growth failure	Yes	NA	NA	Yes (mild)	No	No	No	No	Not available
Weight (SDS)	78 kg (2.03 SD)	NA	NA	13.5 kg (-1.4 SD)	69 kg (+1.06 SD)	13 kg (-0.04 SD)	12y: 43.5 kg (-0.15 SD)	9y: 46 kg (1.88 SD)	NA
Length (SDS)	151.5 cm (-2.6 SD)	NA	NA	92 cm (-1.7 SD)	1.73 m (+0.99 SD)	98 cm (+1.7 SD)	12y: 156.3 cm (-0.01 SD)	9y: 135.5 cm (-0.01 SD)	170cm (+1.03 SD)
OFC (SDS)	53 cm (-1.35 SD)	NA	NA	42.8 cm (-4.2 SD)	55.5 cm (+0.39 SD)	43.5 cm (-2.7 SD)	12y: 51.6 cm (-1.94 SD)	9y: 50.5 cm (-1.51 SD)	51 cm (-3.26 SD)
**Psychomotor development and cognitive function**									
Developmental Delay	Global	Global	Global	Motor	Global, mild	Global	Mild DD	Mild DD	Global
Intellectual disabilty	Severe	Severe	Severe	No, average cognition	Mild	Mild	Mild	Learning difficulties	Mild learning disability
**Neurological**									
Microcephaly (OFC)	No (-1.35 SD)	Yes (NA)	Yes (NA)	Yes (-4.2 SD)	No	Yes (-3 SD)	Yes	No	Yes
Posture	Left sided spastic cerebral palsy	Decerebrate	NA	Could sit and walk with support. Tendon release at 3 y old.	NA	Could sit and stand supported. Hypertonia of lower limbs.	Right sided spastic cerebral palsy	Bilateral spastic cerebral palsy	Generally normal. Mother reports tendency to slump.
Balance	NA	NA	NA	Mildly defective	NA	Defective	NA	Reduced	NA
Spasticity	Yes (unilateral)	Yes (severe)	Yes	Yes, mainly in lower limbs	No	Yes, mainly in lower limbs	Yes (unilateral)	Yes (lower limbs)	No
Hyperreflexia	NA	Yes (severe, with clonus)	Yes (severe, occasional clonus)	Yes in lower limbs	NA	Yes in lower limbs	Yes (unilateral)	Yes (lower limbs)	No
Movement disorders	No	No	Drooling	No	No	No	No	No	No
Abnormal behavior	Anxiety	None reported	None reported	Anxiety/fear (related to intensive physiotherapy and tendon release)	Mild	No	ADHD, anxiety	None reported	Autism spectrum disorder associated with anxiety and mood lability
Sleep disorders/Apnea	NA	Poor sleep	No	No	No	No	Yes	No	No
Additional features	Episodes of psychosis	None	Drooling	Could use the hands well	Asymmetric lower extremities	Drooling, could use the hands but limited fine movement	Urine incontinence, hypercalciuria	Urine incontinence, constipation	Episodes of psychosis
**Epilepsy**									
Seizures	Yes	Yes	No	No	Yes (once)	No	Yes	No	No
Age at onset	6 weeks	15 months	-	-	2 y	-	13 y	-	-
Seizure type	GTCS (single episode)	GTCS	-	-	unknown	-	GTCS	-	-
EEG	NA	Multifocal interictal epileptic discharges, predominant over right hemisphere	NA	Normal	NA	Normal	EEG: Normal. Sleep deprived EEG: Suspicious, not certain epileptic	NA	NA
Seizure response to antiepileptic drugs	Yes	NA	-	-	NA	-	NA	-	NA
Status epilepticus	Yes	NA	-	-	No	-	No	-	No
**Musculoskeletal**									
Fatigability	Yes (with cramping)	NA	NA	Yes	Yes	Yes	Yes	Yes	Yes
Strength	NA	NA	NA	Mild hip girdle weakness	NA	Girdle and lower limb weakness	NA	NA	Non-compliant with examination
Contractures	Achilles contractures	No	No	Tip toeing and tense adductors, requiring tendon release	Tendon release (age 6 months)	No	Achilles tendon - Botox and operation	Achilles tendon. Botox and operation.	No
Other myopathic features	Several episodes of rhabdomyolysis; red ragged muscle fibers	None	None	No	No	No	No	No	No
Lordosis/Scoliosis	Yes (lumbar lordosis)	Yes (lordosis)	No	No	Yes (lordosis and scoliosis)	Mild kyphosis on sitting	No	Yes (lordosis)	No
Skeletal defects	No	None	None	No	Hip dysplasia at birth	Genu recurvatum	No	No	Slim, long-limbed habitus
EMG	Normal	NA	NA	Normal	NA	Normal	NA	NA	NA
**Dysmorphology**									
Facies	Hypotelorism, Broad nasal tip, large upper incisors	Normal	Normal	Normal	Normal	High bossing forehead, sparse thin hair, epicanthic folds, prominent nose, retruded mandible, low set ears	Synophrys, anteverted nostrils, thin upper lip and small chin. Epidermal scar-like nevus left cheek	Anteverted nostrils, thin upper lip, small chin	Normal
**Cardiovascular**									
Blood pressure	NA	Normal	Normal	Normal	Normal	Normal	Normal	Normal	NA
Cardiac abnormalities	No	None reported	None reported	None reported	None reported	None reported	None reported	None reported	Closure of patent ductus arteriosus at age 6 months.
**Sensory**									
Vision, eyes	Normal	Bilateral optic disc pallor, poor vision	Bilateral optic disc pallor	Nystagmus (at 5m)	Normal	Normal	Strabismus, hypermetropia, astigmatism	Hypermetropia	Mild foveal retinal pigment epithelium changes
Hearing	Normal	Normal	Normal	Normal (ABR normal)	Normal	Normal (ABR normal)	NA	NA	Normal
**Additional features**									
Skin	Normal	Normal	Normal	Normal	Cutis marmorata	Large pigmented area in right buttock	Scar-like epidermal nevus left cheek	Normal	Dry skin/dermatitis affecting hands
Other	None reported	None reported	None reported	Tendon release operation	Mild hirsuitism lower legs	None reported	None reported	None reported	Acquired hypothyroidism onset age 13 years
**Neuroimaging**									
White matter signal alterations	Yes, periventricular	No	No	Yes, periventricular	Normal MRI at age 13	Yes, periventricular	Yes, periventricular	Yes, periventricular	NA
Corpus callosum hypoplasia/agenesis	Yes, hypoplasia	Yes, partial agenesis	Yes, partial agenesis	Yes, hypoplasia	No	Yes, hypoplasia	Slightly slender	No	NA
Abnormal cortical gyration	No	Yes, bilateral PMG	No	No	No	No	No	No	NA
Other findings	None	None	None	Small calcifications in the periventricular white matter and right basal ganglia	No	None	Bilateral grey matter calcifications	No	NA

Key
CS = C-section
PROM = premature rupture of membranes
OFC - occipital frontal circumference
GTCS = generalized tonic clonic seizure
NA = not assessed

Supplementary Table 2: Known clinical information for heterozygous relatives of affected individuals

**Initial Report**	This Study	This Study	This Study	This Study	This Study	This Study	This Study	This Study
**Patient ID**	Mother of 1-1	Father of 1-1	Maternal aunt of 1-1	Mother of 2-1 and 2-2	Father of 2-1 and 2-2	Mother of 3-1	Father of 3-1	Mother of 5-1
**Family ID**	F1	F1	F1	F2	F2	F3	F3	F5
**Demographics**								
Year of birth or age at assessment	1974	1973	1973	38 y	40 y	NA	NA	NA
Sex	F	M	F	F	M	F	M	F
Nationality	Norwegian	Norwegian	Norwegian	Pakistani	Pakistani	Egyptian	Egyptian	Egyptian
**Genetics**								
gDNA (hg38)	chr12-21910156-C-T	chr12-21910156-C-T	chr12-21910156-C-T	chr12-21848204-G-A	chr12-21848204-G-A	chr12-21809956-C-A	chr12-21809956-C-A	chr12-21910243-G-A
cDNA†	Het c.1320+1G>A	Het c.1320+1G>A	Het c.1320+1G>A	Het c.2812C>T	Het c.2812C>T	Het c.4212-1G>T	Het c.4212-1G>T	Het c.1234C>T
Protein	p.(Ala389_Gln440del)	p.(Ala389_Gln440del)	p.(Ala389_Gln440del)	p.(Arg938Ter)	p.(Arg938Ter)	p.(Phe1405SerfsTer8)	p.(Phe1405SerfsTer8)	p.(Gln412Ter)
Clinical info	Healthy. No seizures.	Hodgkin lymphoma. Compartment syndrome & rhabdomyolysis.	Premature birth w 29. GTK from childhood, epileptic encephalopathy, mild ID. Has family & children. MRI focal cause of epilepsy, right frontal lobe	Healthy	Healthy	Healthy	Healthy	Healthy; reported repeated unexplained abortions
Hypertension	NA	NA	No	No	No	No	No	No

**Initial Report**	This Study	This Study	This Study	Smeland et al 2019	Smeland et al 2019	Smeland et al 2019	Smeland et al 2019	Smeland et al 2019
**Patient ID**	Father of 5-1	Father of 6-1 and 6-2	Mother of 6-1 and 6-2	Father of Family 1 in Smeland et al 2019	Mother of Family 1 in Smeland et al 2019	Aborted fetus of Family 1 in Smeland et al 2019	Father of Family 2 in Smeland et al 2019	Mother of Family 2 in Smeland et al 2020
**Family ID**	F5	F6	F6					
**Demographics**								
Year of birth or age at assessment	NA	1982	1988	1968	1975	Not relevant	1961	1966
Sex	M	M	F	M	F	F	M	F
Nationality	Egyptian	Norwegian	Norwegian	Norwegian	Norwegian	Norwegian	Norwegian	Norwegian
**Genetics**								
gDNA (hg38)	chr12-21910243-G-A	chr12-21910156-C-T	chr12-21910156-C-T	chr12-21910156-C-T	chr12-21910156-C-T	chr12-21910156-C-T	chr12-21910156-C-T	chr12-21910156-C-T
cDNA†	Het c.1234C>T	Het c.284+1G>A	Het c.284+1G>A	Het c.1320+1G>A	Het c.1320+1G>A	Het c.1320+1G>A	Het c.1320+1G>A	Het c.1320+1G>A
Protein	p.(Gln412Ter)	p.(Phe49GlyfsTer13)	p.(Phe49GlyfsTer13)	p.(Ala389_Gln440del)	p.(Ala389_Gln440del)	p.(Ala389_Gln440del)	p.(Ala389_Gln440del)	p.(Ala389_Gln440del)
Clinical info	Healthy	Healthy	Healthy	Morbus Bechterew. Ulcerous colitis. Normal cardiac evaluation at age 51.	Healthy. Normal cardiac evaluation at age 46	"Thanatophoric dysplasia" found in pregnancy - terminated. No FGFR3 mutation.	Dilated cardiomyopathy from age 60	Normal cardiac evaluation at age 51
Hypertension	No	NA	NA	No	No	Not relevant	Yes	No

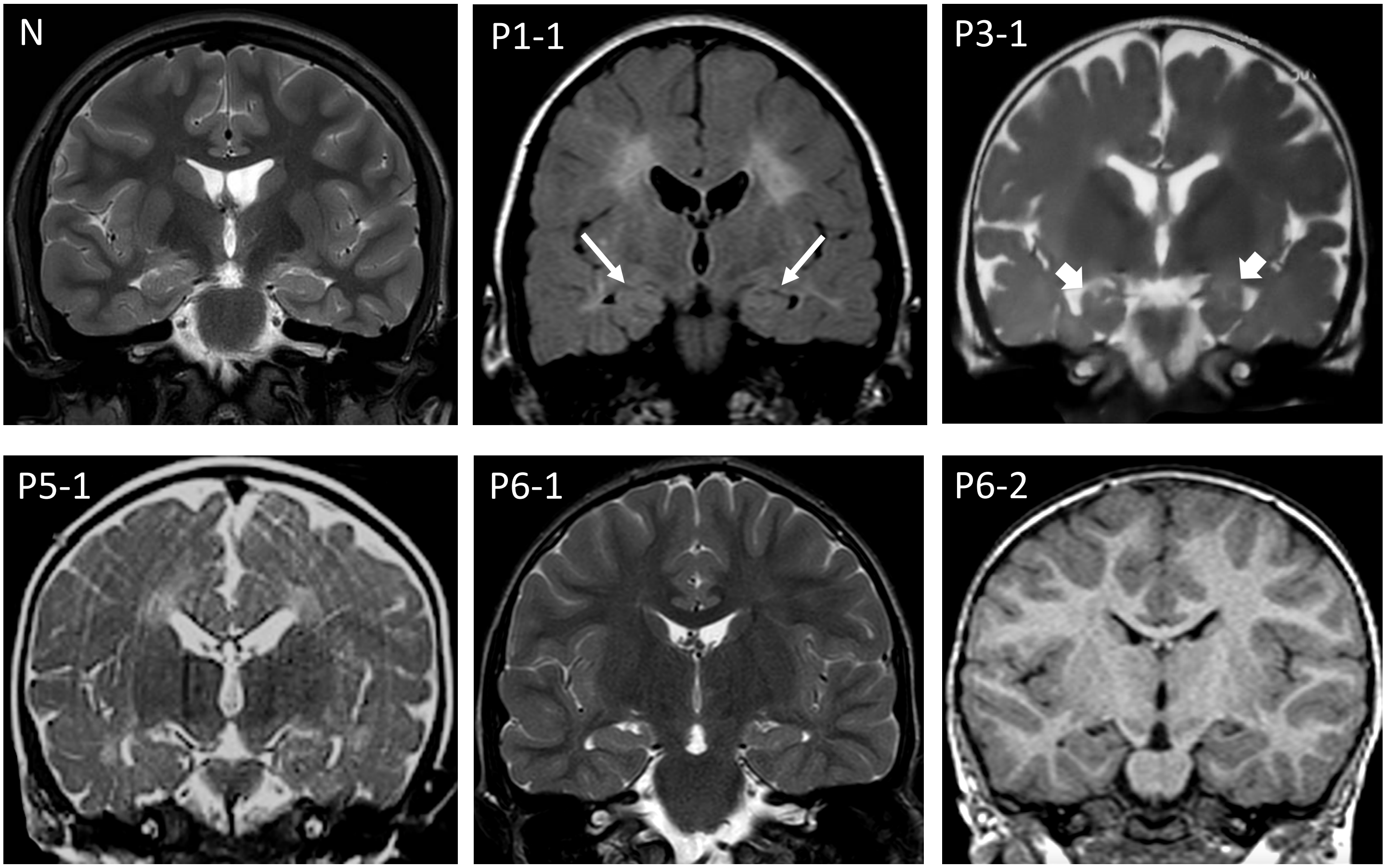

Supplementary Figure 1

Hippocampal features in subjects with AIMS and a normal control for comparison. Brain MRI with coronal T2-weighted, FLAIR or T1-weighted images demonstrate slightly smaller volume of the hippocampi in subject P1-1 (thin arrows) and incomplete hippocampal rotation in subject P3-1 (thick arrows). No signs of hippocampal sclerosis are present.

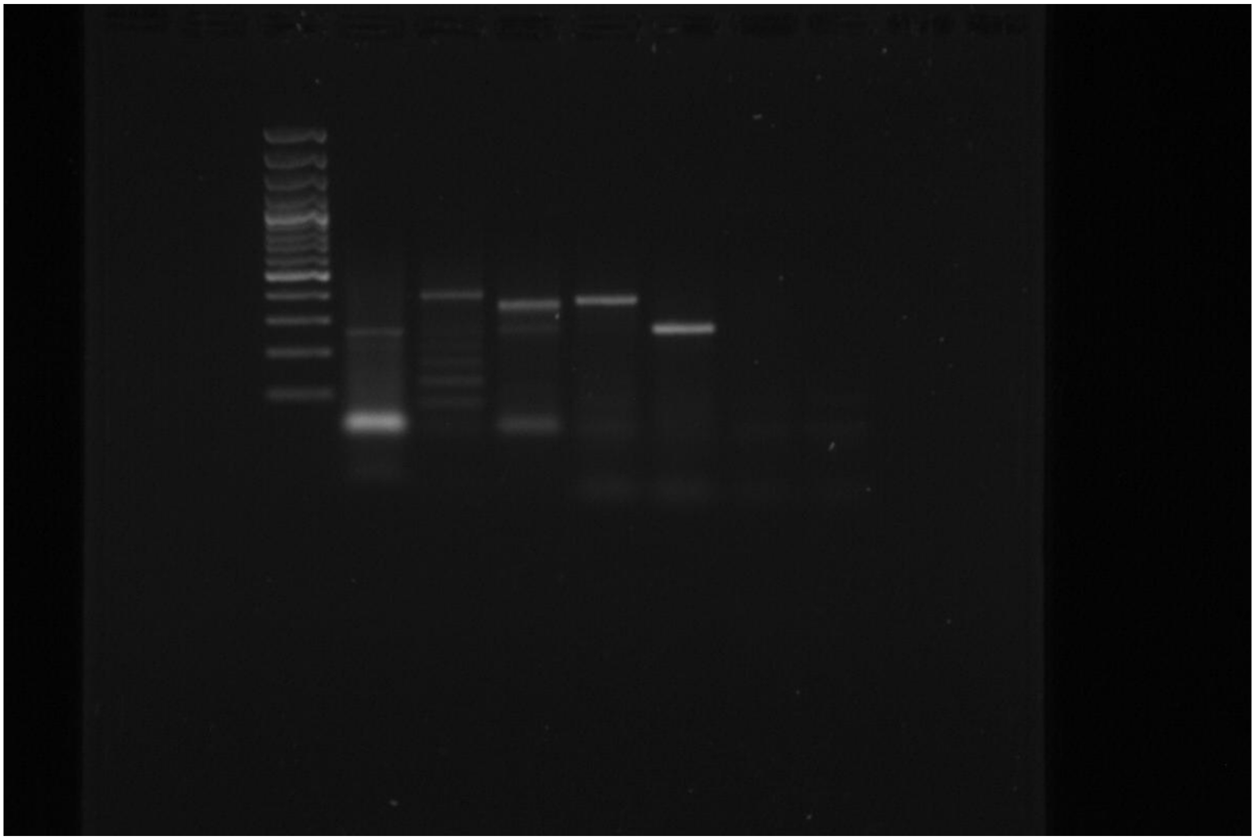

Uncut gel image from Figure 2A
